# Association Between Simian Virus 40 and Human Tumors

**DOI:** 10.3389/fonc.2019.00670

**Published:** 2019-07-25

**Authors:** John Charles Rotondo, Elisa Mazzoni, Ilaria Bononi, Mauro Tognon, Fernanda Martini

**Affiliations:** Section of Pathology, Oncology and Experimental Biology, Department of Morphology, Surgery and Experimental Medicine, University of Ferrara, Ferrara, Italy

**Keywords:** simian virus 40, polyomavirus, cancer, tumor, malignant pleura mesothelioma, osteosarcoma, healthy subjects, ELISA

## Abstract

Simian virus 40 (SV40) is a small DNA tumor virus of monkey origin. This polyomavirus was administered to human populations mainly through contaminated polio vaccines, which were produced in naturally infected SV40 monkey cells. Previous molecular biology and recent immunological assays have indicated that SV40 is spreading in human populations, independently from earlier SV40-contaminated vaccines. SV40 DNA sequences have been detected at a higher prevalence in specific human cancer specimens, such as the brain and bone tumors, malignant pleural mesotheliomas, and lymphoproliferative disorders, compared to the corresponding normal tissues/specimens. However, other investigations, which reported negative data, did not confirm an association between SV40 and human tumors. To circumvent the controversies, which have arisen because of these molecular biology studies, immunological researches with newly developed indirect ELISA tests were carried out in serum samples from patients affected by the same kind of tumors as mentioned above. These innovative indirect ELISAs employ synthetic peptides as mimotopes/specific SV40 antigens. SV40 mimotopes do not cross-react with the homologous human polyomaviruses, BKPyV, and JCPyV. Immunological data obtained from indirect ELISAs, using SV40 mimotopes, employed to analyze serum samples from oncological patients, have indicated that these sera had a higher prevalence of antibodies against SV40 compared to healthy subjects. The main data on (i) the biology and genetics of SV40; (ii) the epidemiology of SV40 in the general population, (iii) the mechanisms of SV40 transformation; (iv) the putative role of SV40 in the onset/progression of specific human tumors, and (v) its association with other human diseases are reported in this review.

## Introduction

Simian virus 40 (SV40) is a monkey virus that was accidentally administered to human populations through SV40-contaminated vaccines, mainly polio vaccines, between 1955 and 1963 (1). SV40 has been assigned to the family of *Polyomaviridae*, Betapolyomavirus genus, which is closely related to human JC (JCPyV) and BK (BKPyV) polyomaviruses (HPyVs) (2). Many studies have reported on the transforming and tumorigenic properties of SV40, which have been experimentally proven in cell cultures and animal models, respectively (3–7). These data have encouraged a significant amount of new researches aimed developed aimed at verifying if an association between SV40 and different human cancers exists.

This review provides a brief overview on the (i) biology and genetics of SV40; (ii) the epidemiology of SV40 in the general population, (iii) the mechanisms of SV40 transformation; (iv) the putative role of SV40 in the onset/progression of specific human tumors, and (v) its association with other human diseases.

## SV40 Genomic Organization

The SV40 virion is formed by an unenveloped icosahedral protein structure with a diameter of 45–50 nm and a density of 1.34–1.35 g/cm^3^ (8). Its viral genome is a circular double-stranded DNA molecule with ~5.2 kb, depending on the SV40 strain (9). SV40 shares about 70–75% genome homology with JCPyV (10–12) and BKPyV (12, 13), whereas it has little homology with other HPyVs, including HPyV 6 and 7 (14), Malawi polyomavirus (MXPyV or HPyV 10) (15), Saint Louis polyomavirus (STPyV or HPyV 11) (16), and Merkel cell polyomavirus (MCPyV) (17–19).

**Figure 1:**
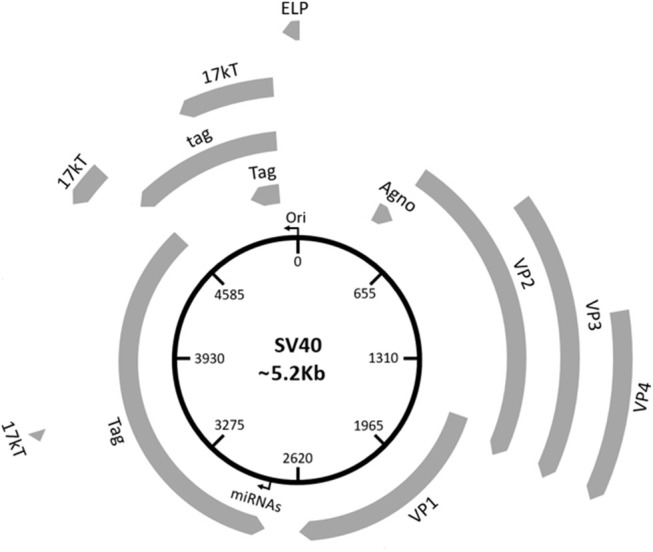
Schematic representation of the SV40 genome. SV40 DNA is made up of three regions: the regulatory region and the early and late regions. The regulatory region contains the origin of replication (*ori*) and regulates the viral gene expression. The early region contains coding sequences for early genes, including the large tumor T antigen (Tag), the small tumor t antigen (tag), 17 kT, and the early leader protein (ELP). The late region contains coding sequences for late genes, including the major capsid protein VP 1, the VP 2, VP 3, VP 4, and the agnoprotein (Agno). The two miRNAs maps within the Tag gene sequences.

**Figure 2:**
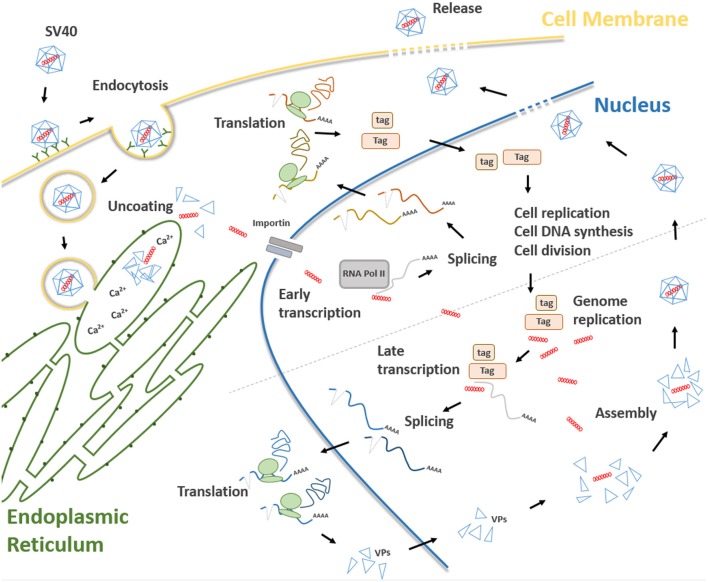
Main steps of the SV40 life cycle. The life cycle of SV40 starts with the attachment of the SV40 viral capsid to the target cell surface and proceeds through a lipid raft-mediated endocytosis. Then, the virion is transferred, by vesicular transport, toward the endoplasmic reticulum where it starts the uncoating process which continues in the cell cytosol. Uncoated SV40 genomes translocate inside the nucleus where the cellular RNA polymerase II mediates early viral transcription. Early transcription generates a precursor that is alternatively spliced into mRNAs, encoding the large T (Tag), and small t (tag) antigens. These mRNAs are translated in the cytosol into their corresponding proteins. Tag and tag migrate to the nucleus where they mediate several functions interfering with a number of host cellular pathways, thereby forcing the cells to proceed from the G1 to the S-phase. At the same time, Tag/tag starts the replication of the SV40 genome. The transition from early to late phase during the SV40 infection begins at the end of the viral DNA replication. After synthesis, late viral proteins are accumulated in the cytoplasm, migrates into the nucleus and then assemble with replicated viral DNA to form virions. Finally, a progeny virus is released through cell lysis.

Three main regions have been identified in the SV40 genome: (i) a non-coding control region (NCCR), (ii) an early, and (iii) late coding regions (Figure 1). NCCR includes the DNA replication origin (ori) and a gene promoter, whose nucleotide sequences are binding sites for transcription factors regulating early and late gene expressions. The terms “early” and “late” indicate the chronological order of gene transcriptions during the viral life cycle in the host cell. Both early and late genes are transcribed in opposing directions, i.e., anti-clockwise and clockwise, respectively, in relation to NCCR. The early region contains coding sequences for the large tumor antigen (Tag), small tumor antigen (tag), 17 kT and the early leader protein (Figure 1, Table 1). Both Tag and tag are transcribed with alternative splicing. Tag and tag are viral oncoproteins, which induce SV40 DNA replication, gene expression, as well as S-phase entry and DNA synthesis in the host cell, thereby triggering cycle progression (Figure 2) (9). In addition, these two oncoproteins own transformation potential *in vitro* and exert oncogenic activities *in vivo* (9). The 17 kT protein, which shares most of its amino acid (aa) sequence with the Tag N-terminal domain, promotes cell cycle progression in the presence of tag, as well as presenting tumorigenic potential (20). The early leader protein is a small protein of 23 aa whose function is unclear (21). The late region contains genes transcribed into two classes of late mRNAs: (i) 16S, which encodes the major capsid protein VP 1; (ii) 19S, coding for the VP 2, VP 3, VP 4 polypeptides, and the agnoprotein (Figure 1, Table 1). VP 1-2-3 are structural proteins that enable viral DNA to be packaged into the SV40 virion. A total of 360 VP 1 molecules form, with 72 pentamers, the virion (22, 23). The internal face of each pentamer binds a single copy of VP 2 or VP 3 (22). VP 4 seems to facilitate the lytic release of the SV40 virions (24), but a recent study demonstrated that VP4 is not required for this process (25). The agnoprotein controls the perinuclear localization of VP 1 during virion formation, which then triggers virion assembly (26). In total, SV40 translates for nine proteins. Recently, two SV40-encoded microRNAs (miRNAs) have been identified (Figure 1, Table 1) (27). More details are reported on this topic in the section “SV40 microRNAs and viral infection” (see below).

**Table 1:** SV40 gene products.

	**SV40 expression products**	**Main function(s)**
Early	Large tumor T antigen (Tag)	Cell cycle progression, viral DNA replication
	Small tumor t antigen (tag)	Cell cycle progression, viral DNA replication
	17 KT	Cell cycle progression
	Early leader protein	Unclear function
	Early polarity SVmiRNA	Tag regulation
	Late polarity SVmiRNA Tag regulation	
Late	VP1	Capsid structure (external), viral attachment and entry
	VP2	Capsid structure (internal)
	VP3	Capsid structure (internal)
	VP4	Cell lysis, viral particles release
	Agnoprotein	Virion assembly

## SV40 Life Cycle

Attachment of the SV40 viral capsid to the target cell surface is the first event to take place during the infection process in host cells. Binding is mediated by an interaction between VP 1, the cell surface receptor ganglioside GM1 (28), and the major histocompatibility complex (MHC) class I, that act as co-receptors (29). Afterwards, SV40 capsid enters the target cell via lipid raft-mediated endocytosis (30), which is triggered by the interaction between VP 1 and cell surface ganglioside GM1 (31) (Figure 2). Then, SV40 capsid is transferred, by vesicular transport, in the endosomal compartment, toward the endoplasmic reticulum (ER). In atypical circumstances, SV40 enters the cells via a caveolae-mediated endocytic pathway by which the virus is directly translocated to the ER (32, 33), bypassing the endosomal compartment. The uncoating process begins in the ER, proceeds through ER absorption of Ca^2+^ ions, thus inducing the loss of specific inter-pentamer connections provided by invading VP 1 C-terminal arms in the capsid. This process exposes the SV40 nuclear localization signal, thereby inducing the translocation of the viral genome in the nucleus via a mechanism mediated by the importin α_2_/β heterodimer and VP 3 (8, 34, 35) (Figure 2). Both early viral transcription and DNA replication occur inside the nucleus. Transcription is NCCR regulated (36), whereas DNA replication starts from the ORI sequence contained in the same NCCR region. SV40 DNA replication occurs soon after transcription in the early region, whereas late region transcription initiates after replication of viral DNA (37). Both early and late promoters are recognized by cellular RNA polymerase II and host factors, thereby inducing viral gene transcription. Early transcription provides the generation of a precursor that is alternatively spliced into two mRNAs encoding Tag and tag (38) (Figure 2). In this phase, SV40 late genes are maintained silenced by transcriptional repressors (39). In permissive cells, the role of Tag is essential for DNA replication. Tag is a multifunctional phosphoprotein that binds as a double hexamer to the SV40 viral replication ORI, where it unwinds viral DNA. This molecular process induces cellular protein recruitment required for viral DNA replication, including DNA polymerase-α and replication protein A (40, 41). Tag is also responsible for an ATPase activity that is required for viral DNA elongation (42). SV40 needs additional cellular co-factors for its DNA replication, mainly expressed during the S phase. For this reason, Tag is evolutionarily developed to modulate intracellular proteins involved in crucial signal transduction pathways that control cell cycle progression and apoptosis (43), such as hepatocyte growth factor receptor (HGFR/Met) (44), insulin-like growth factor 1 (IGF-1) (45), Notch-1 (46), and cdc2 (47). These molecules force SV40-infected cells to proceed from the G1 to the S-phase (48). In this mechanism, tag seems to play a cooperative role with Tag in both SV40 DNA replication and S-phase progression (Figure 2) (7, 49, 50). The transition from the early to late phase, during the SV40 infection, begins at the end of the viral DNA replication. It seems that this early-to-late transcriptional switch depends on changes on Tag concentration. Initially, low Tag concentrations are sufficient for an interaction between high-affinity NCCR Tag-binding motifs and Tag and thus early transcription is positively regulated. Then, high Tag concentrations enable this protein to interact with low-affinity NCCR Tag-binding motifs. This interaction induces the repressing early transcription by blocking the RNA polymerase II complex. In addition, cellular repressors are titrated-off the late promoter allowing the expression of late genes. Indeed, since the number of SV40 genomes increases during viral DNA replication, the concentration of repressors is reduced in the late promoter. Tag, together with host transcription factors, interacts with the late promoter, thereby inducing late gene transcription (51). Late genes are transcribed in an opposite direction to the early gene-encoding strand. Late proteins are translated from two classes of late mRNAs: (i) 16S, which encodes the major capsid protein VP 1; (ii) 19S, coding for VP 2, VP 3, VP 4 polypeptides, and agnoprotein (52) (Figure 2). After synthesis, late viral proteins are accumulated inside the cell via checkpoint kinase Chk1 activation by SV40, which negatively regulates cell mitosis (53). Then, structural proteins assemble with replicated viral DNA to form virions inside the nucleus (Figure 2) (26). This mechanism is induced by the six tandem GC-boxes within the SV40 genome, which represent the capsid assembly signal. Viral assembly starts with GC-boxes interacting with cellular transcriptional factor SP1 recruiting VP 2 and VP 3, which in turn bind to VP 1 pentamers (54). During this process, the number of capsomers surrounding the viral DNA increases until virion assembly has ended (9). At the same time, the agnoprotein controls perinuclear VP 1 localization (26). Then, the viral particle releasing (Figure 2) leads to cell lysis and necrosis. However, the release of SV40 without displaying a cytopathic effect (CPE) has been reported in specific cell types, such as human mesothelial, epithelial, fibroblasts, and/or embryonic kidney cells (HEK) (44, 55–57).

## SV40-Mediated Cell Immortalization and Transformation

Mammalian cells of different histotypes behave toward SV40 infection in different ways, depending on the ability of this oncogenic polyomavirus (PyV) to complete the viral cycle and produce a mature viral progeny. SV40-infected cells can be (i) permissive, (ii) non-permissive, or (iii) semi-permissive (56, 58–60). The main discriminant depends on viral DNA replication potential expressed in permissive and semi-permissive cells. In this case, viral progeny is produced, whereas SV40 infected cells lyse and die. CV-1 and fibroblast-like COS cell lines, both derived from monkey kidney tissue, are the prototype of permissive cells (58, 61). In non-permissive cells, no productive viral cycle is established, whereas the infection occurs but is abortive. Indeed, these cells are transformed/immortalized by SV40. A typical example of non-permissive cells are rodent cells, that carry SV40 DNA integrated in their genome, while cells are transformed (62). Semi-permissive cells allow SV40 multiplication, but they produce a limited viral progeny (63). The majority of cells lyse and die upon infection, but a fraction of cells, which resist the SV40 infection, are transformed and immortalized, while producing a viral progeny at low titer. Several SV40 transformed/immortalized human fibroblasts have been described in the literature (64, 65). Cells differ in response to SV40 infection depending on the ability of Tag to stimulate late promoter transcription, which only occurs in permissive/semi-permissive cells. Human cells support SV40 replication less efficiently than monkey cells. Different *in vitro* cellular models have been established to demonstrate the replicative potential of SV40 in human cells (66). Early studies have shown that SV40 can replicate in human fetal neural cell lines (4), mesothelial cells (56), and B-/T-lymphocytes (64, 65, 67). Although less efficiently, this PyV can also replicate in human HEK lymphoblastoid B-cell lines, as well as fibroblasts, such as WI-38 cells (57, 68–71). In addition, in rare cases (<1/10^8^ cells), human fibroblasts may become transformed due to the viral DNA integration in the host cell genome (72). Early works have shown that one out of seven human astrocytes could become transformed (73) establishing continuous cell lines (74). In addition, several SV40 infected human cells produce a viral progeny at low titer without displaying CPE (44, 56, 57). An example is provided by normal human mesothelial cells (HMC), which seem to be persistently infected by SV40 for a long period of time, while releasing viral progeny (44, 56, 57). The molecular mechanism behind the capacity of SV40 to enter into a true persistent/latent state remains to be fully elucidated. It has been reported that SV40 is able to establish a persistent infection in long-term immortalized human fibroblasts, resulting in the production of infectious viral progeny, which is able to infect both monkey and human cells (64, 65).

**Figure 3:**
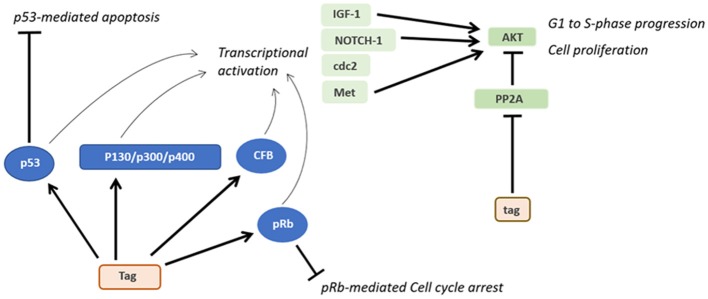
Oncogenic activities of Tag and tag. During SV40 infection, Tag inhibits both the pRB and p53 tumor suppressor pathways. The main downstream effects of these interactions are the blocking of p53-mediated apoptosis and pRb-mediated cell cycle arrest. Furthermore, the interaction between Tag and pRB, p53 and other factors transactivates genes such as IGF-1 and Met, thereby triggering the transition from the G1 to the S-phase and the proliferation of SV40-infected cells. By inhibiting PP2A, tag activates pathways facilitating cell proliferation and transformation.

As for other DNA tumor viruses (75, 76), several *in vitro* cellular models have been developed to study SV40 transformation potentials. Tag and tag expression both cause high cell transformation efficiently (3). Indeed, Tag blocks the activities of many different cellular factors involved in cell growth, differentiation and the cell cycle, such as p130, p300, and p400 (Figure 3) (9). In addition, both Tag and tag can inhibit the activities of p53 and pRb, which are two key tumor suppressor proteins of animal and human cells (9). These interactions are mandatory in order to achieve full human cell transformation (9). Similarly, the transformation potential of Tag belonging to another PyV, MCPyV, has been demonstrated. Indeed, MCPyV Tag or LT has been detected to be overexpressed on MCPyV-positive tumor tissues (17) and several *in vitro* studies evaluated its transforming activity (77, 78). However, it is important to point out that many studies, while reporting the presence of SV40 sequences in human tumors, did not show the Tag expression. On the other hand, in investigations based on IHC staining of human tumor tissues, the Tag expression was revealed in SV40-positive cells (79–85).

Interestingly, p53 was discovered for the first time when detected bound to Tag in SV40-transformed cells during immunoprecipitation experiments (86). Both Tag and tag can interfere with many other host cellular pathways (38, 87, 88). For instance, Tag has been found to be associated with many cellular factors, such as Hsc70, Cul7, Bub1, TEF-1, Nbs1, and Fbw7 (59). It has also been reported that the Tag-p53 complexes bind and activate IGF-1 promoter stimulating cell growth (45). The expression of other growth factors may be potentially influenced by Tag (9). Another cellular factor that interacts with Tag during SV40 infection is the hepatocyte growth factor receptor (HGFR or Met) (44). Furthermore, Tag is capable of inducing cell immortalization and transformation by increasing CBP/p300 and specific histone acetylation levels (89).

Upon interaction with the cellular genome and cell factors, SV40 Tag induces different molecular changes in the host cell. SV40 Tag possesses clastogenic and mutagenic activities, which are shown by the appearance of chromosome aberrations and point mutations in the host genome (90–93). These molecular/chromosome changes have not been investigated/reported in human tumors presumably caused by SV40. Specific genome alterations are characterized by numerical and structural chromosomal aberrations, such as gaps, breaks, dicentric and ring chromosomes, chromatid changes, deletions, duplications, and translocations (91–93). SV40 Tag favors the accumulation of point mutations, interfering with host DNA repairs pathway (90, 94, 95). Indeed, SV40 Tag binds p53 protein thereby inhibiting p53-induced apoptosis and allowing DNA-mutated cells to survive (90, 94, 95). It is well-established that specific chromosomal aberrations can be detected in human tumors of different histotypes, as reported in: (http://atlasgeneticsoncology.org/Deep/CancerCytogenet2005ID20050.html). In SV40-driven/positive human tumors, a similar panel of chromosome alterations could be displayed (90–93). Occasionally, SV40 DNA integrates into the host cellular genome. This event can occur randomly in the cellular DNA, while the viral DNA breaks randomly, as well (96). In cases where SV40 DNA integration occurs, while maintaining Tag and tag expression, these two viral oncoproteins support the cell transformation phenotype (9). In addition, Liu and collaborators demonstrated that the disruption of the human chromosomal interval at 1q21.1 by SV40 integration, in the human bronchial epithelial cell line, immortalized with a cloned Ori– SV40, CRL-2504 cell line, can be an essential step for cellular immortalization, altering the expression of genes involved in senescence/apoptosis, which are located in the proximity of viral integration sites (97). Integration of SV40 DNA within the human genome has been reported in osteosarcoma samples, thus suggesting that the viral DNA integration is involved the tumorigenic process (98). Altogether these SV40-driven alterations represent the genetic background, which drives the phenotypical changes that lead to the SV40-induced transformation process of the host cell.

The small tag viral oncoprotein interacts with the protein phosphatase 2A (PP2A), thereby triggering several pathways related to cellular transformation (Figure 3). The complex tag-PP2A induces the entry on the cell cycle S-phase through CDK inhibitor p27 degradation and cyclin A/CDK2 and cyclin E/CDK2 promotion (99–101). Furthermore, tag-PP2A induces MAPK cascade activation, thereby triggering the transforming process *in vitro* (102). The third SV40 early protein, 17kT, also transactivates the cyclin A/CDK2 (100). Although many factors responsible for SV40 transformation have been discovered, the total number of proteins involved in this activity is yet to be determined.

An association between SV40 infection and DNA methylation of different cellular genes has been reported. Indeed, improper DNA methylation is involved in different diseases (103, 104), including cancer (105, 106). DNA methylation is induced by DNA tumor viruses in order to evade antiviral immunity, which contributes to the immunosuppressive microenvironment during cancer development (107). This process facilitates viral multiplication/activity (107).

In early studies, SV40 transforming potential was largely employed in developing different *in vitro* cellular models. SV40-immortalized cells have been established and used to study a large number of different molecular mechanisms, including cell proliferation and transformation (108–110), cytokine-production (111), and angiogenesis (112), as well as mesenchymal stem cell differentiation (113) and neuronal differentiation and neuroregeneration (114). Moreover, different diseases, such as autoimmune disorders (115), male infertility (116), fibrosarcoma (108), and corneal dystrophy (117) have been studied employing SV40-immortalized cell models. Other applications of SV40-immortalized cell *in vitro* models are represented by cellular co-culturing (118) and suicide gene therapy (119).

## SV40 MICRORNAs and Viral Infection

MicroRNAs (miRNAs) are small non-coding RNAs [18–22 nucleotides (nt)] that are involved in the post-transcriptional negative regulation of gene expression in eukaryotes (120). These small molecules and their regulatory effect have been described in both eukaryote cells and viruses, including PyVs (120). In the SV40 the early region maps a gene encoding for two miRNAs (Figure 1), which are transcribed in opposite orientations. These two viral miRNAs negatively regulate early mRNAs inhibiting Tag translation through RNA-mediated interference (RNAi) machinery, during the late phase of the SV40 life cycle. These two miRNAs actively direct Tag mRNA cleavage at different nucleotide positions (27, 121). However, the silencing potential of human genes by SV40 miRNAs cannot be ruled out (27, 121). These findings indicate that SV40 is able to use human RNAi machinery to its own advantage. Since Tag is a target for host cytotoxic T lymphocyte (CTL), the viral miRNA-mediated down-regulation of Tag decreases the susceptibility of SV40-infected cells to CTLs activity (27, 121). Similarly, JCPyV and BKPyV escape the innate and adaptive immune detection exploiting the human RNAi machinery. Indeed, these two HPyVs code for a miRNA identical in sequence between BKPyV and JCPyV, which targets a member of the UL16 binding protein (ULBP) family, the stress-induced ligand ULBP3 (122). Although a recent study indicates that another member of this gene family, ULBP1, is down-regulated following SV40 infection, it has been demonstrated that SV40 miRNAs do not mediate this molecular effect, thus suggesting the involvement of other mechanisms behind the SV40 immune evasion ability (123). Another report showed that the two SV40 miRNAs can negatively regulate the degree of viral effects on B-cells as demonstrated using SV40 miRNA-null mutants in experiments with infected B-lymphocytes and myeloid cell lines (124).

## SV40 Oncogenicity in Animal Models

SV40 is a high oncogenic small DNA tumor virus. However, this PyV has not been reported in tumors of the rhesus macaque (Macaca mulatta), which is its natural host. Indeed, SV40 infection in permissive monkey cells derived from kidney tissues leads to cell lysis and death, without neoplastic transformation (58, 61). By contrast, SV40 experimentally inoculated in hamsters induces tumors of different histotypes, depending of the route of injection. Specifically, SV40 subcutaneously (s.c.) inoculated in hamsters induces sarcomas and osteosarcomas; whereas when injected intracerebrally (i.c.) it induces ependymomas and choroid plexus papillomas (5, 38). Hematological malignancies, such as lymphocytic leukemia, histiocytic lymphomas, and B-cell lymphomas are induced when SV40 is inoculated intravenously (i.v.) (5, 6). SV40 injected in the pleural space of hamsters induces MPM in 100% of animals (125).

SV40 oncogenic activity is shown in transgenic mice, where Tag/tag expression is under the control of its promoter (126) or tissue-specific promoters (127–145). Exploiting the vast SV40 oncogenicity *in vivo*, SV40 transgenic animals provided good models for studying tumor initiation/progression and innovative anti-cancer therapies (146). Transgenic animals develop ependymomas, choroid plexus papillomas (147), hepatocellular carcinomas (139, 144), brain (148), bladder (140), and bowel tumors (149), as well as eye tumors, including ocular and/or lens tumors (150). The multistage progression of prostate carcinoma has also been largely studied employing SV40-transgenic mice (128–130, 132, 134–136, 151, 152). Interestingly, transgenic mice generated with SV40 have also been employed to study rare cancers, such as brown adipose tumor (151), cardiac rhabdomyosarcoma (142) and adrenocortical carcinoma (151). Lung cancers and MPM have also been studied in these animal models (127, 137, 141). More recently, SV40-transgenic mice have been developed to study chronic lymphocytic leukemia (143). In addition, other SV40-transgenic mouse models have also been developed to study fibrosarcoma (108) and retinoblastoma (153) as well as breast (154, 155) ovarian (131, 156, 157), pancreatic (158), and liver cancers (20, 159).

## Epidemiology of SV40 Infection in Human Populations

SV40 infection in human populations has been widely reported (160–164). Since SV40 is a monkey virus and the macaque is its natural host, this viral infection in humans was considered a rare event, being restricted to subjects in close contact with monkeys. Indeed, inhabitants of Indian villages located near the jungle, which is the natural environment for monkeys, and workers attending to monkeys in zoos/animal facilities are prone to SV40 infection and develop antibodies against this PyV (165, 166).

SV40 was inadvertently administered to humans between 1955 and 1963, when hundreds of millions of people in North and South America, Canada, Europe, Asia, and Africa were vaccinated with both inactivated and/or live polio vaccines, found to be contaminated by SV40. This accident occurred because these early polio vaccines were produced by growing polioviruses in naturally SV40-infected monkey cell cultures (167–169). It has been reported that in the former USSR, SV40-contaminated polio vaccines were used until 1978 (170), whereas in Italy up to 1999, when the Italian Health Public Organization switched to SV40-free anti-polio vaccines as indicated by the World Health Organization (WHO) guidelines, following a note from the British National Institute for Biological Standards and Control (9, 74, 171). In other countries, the risk of SV40 contagion through polio vaccines is still a problem, as these vaccines are produced using SV40-positive monkey cells (170). The past literature indicates that SV40 infection in different geographic regions was influenced by the use of either SV40-contaminated or non-contaminated vaccines, as well as the number of years of vaccine administration. Sweet and colleagues quantified that about 10–30% of polio vaccines were contaminated with SV40 (1). Furthermore, SV40 genotypes in polio vaccines overlap with those detected in humans, thus suggesting that this oncogenic PyV was introduced into the human population through contaminated polio vaccines (172). It has also been reported that shortly after SV40 infection, this PyV spread for weeks in the stools of children vaccinated with SV40-contaminated vaccines (173). This evidence indicates that SV40 replicates in some gastrointestinal cells, thus suggesting that this virus could spread in humans via horizontal infection, such as the fecal-oral route. To a lesser extent, other vaccines against adenoviruses (174) and hepatitis A (175), were SV40 contaminated. In addition, SV40-contamination was detected in the respiratory syncytial virus vaccine employed in infected-volunteers to whom the vaccine was given to via the respiratory route (176). About two out of three volunteers developed neutralizing antibodies against SV40 (176). Altogether these data indicate that SV40 infects and multiplies in humans.

Over the years, with the development of molecular biology techniques (12, 18, 76, 177, 178), SV40 DNA sequences have been investigated and detected in both normal and neoplastic tissues from individuals vaccinated with polio vaccines contaminated with SV40. DNA sequences from this PyV have been detected in pituitary tissues (179) as well as in leukocytes from organ (180) and blood (181) donors. Footprints from SV40 DNA have also been reported in lymphoblastoid cells (68), as well as blood sample specimens from normal individuals and oncologic patients (79, 161, 182–186). In addition, SV40 DNA has been detected in blood samples from healthy individuals exposed to asbestos pollution (187). These data cumulatively demonstrate that SV40 is circulating in the human population. It is also possible that blood cells are the SV40 reservoir and vehicle of the virus spreading in humans. Genomic sequences from this PyV have also been found in stool samples and urine from children and adults, suggesting that SV40 can potentially be transmitted via different routes, such as sexual and fecal-oral routes which are responsible for viral horizontal infection in humans (164, 183, 188). These additional sources of exposure may lead to subclinical SV40 infections in the healthy population. However, it has been reported that the SV40 transmission in monkeys seems to occur in the environment rather than directly among animals (189). It is plausible that in humans, a contaminated environment or home setting is responsible for SV40 spreading, rather than person to person transmission (188). The site of SV40 latent infection in humans is yet to be elucidated. Since this PyV has been detected in human kidney and urine samples (183, 184) it seems reasonable that kidneys might be the site of virus latency, as in monkey it occurs to be the natural host (1, 189).

SV40 primary infection occurs early in life and its seroprevalence increases with age. Anti-SV40 antibodies in the serum of immunized individuals and SV40 antigens have been detected in normal subjects (190–199). Lusting et al. reported a prevalence of serum anti SV40 antibodies in 7.6–14% of Swedish children aged from 1 to 13 years old (198). In early studies, a low prevalence, 11%, was detected in healthy individuals from Africa (200) and the U.S (201). Similarly, a more recent study detected anti SV40 antibodies in sera from healthy adult blood donors with low rates, about 2% (202). Altogether these serological data indicate that SV40 is present in immunized healthy populations in the range of 1.3–15.6%, suggesting that this PyV circulates in humans at low prevalence (200, 201, 203–206). Interestingly, one study reported SV40 antibodies in human sera before the introduction of SV40-contaminated polio vaccines, suggesting that SV40 was probably circulating in humans independently from SV40-contaminated vaccines (207). However, this study was conducted before the identification of human PyVs, JCPyV, and BKPyV, which are highly homologous to SV40 (2, 13). Indeed, the high homology among the three PyVs, BKPyV, JCPyV, and SV40, hampered serological data due to antigenic cross-reactivity. For many years, PyV cross-reactivity did not allow the verification of the real SV40 prevalence in humans (208). Different immunological assays based on the use of virions, soluble recombinant VP 1 protein and virus-like particles (VLPs), such as SV40 antigens, always gave cross-reactivity with BKPyV/JCPyV (208).

In order to circumvent this technical issue, in recent years specific and sensitive indirect ELISA tests with SV40 VPs/Tag mimotopes as antigens were set up to investigate the presence in healthy subjects of serum IgG/IgM antibodies reacting to SV40 VPs/Tag (191, 192). Immunological data showed that healthy blood donors carry IgG antibodies reacting to SV40 VPs and Tag with a prevalence of 18 and 20%, respectively (191, 192). Furthermore, immunological data from children may suggest that SV40 infection/seroconversion occurs early in life, i.e., at 6 months of age (190, 193).

Antibodies against SV40 VPs in sera from multiple transfused patients affected by thalassemia major had a higher prevalence than healthy subjects of the same age (31 years old, both cohorts). These data indicate that this PyV could have been transmitted by blood transfusions, along with other natural sources (209). Furthermore, the increased prevalence of SV40 antibodies was significantly higher in the older age group of patients (41–50 years old) than in age-matched controls (38 vs. 20%).

In a recent investigation, SV40 neutralizing antibodies with a prevalence in the range of 7 and 18% were revealed in sera from women in Huston, Texas, employing a plaque reduction SV40-neutralization assay. The authors identified ethnicity as a significant factor associated with high seroprevalence SV40 neutralizing antibodies reported in Hispanic groups, including subjects from Houston (210). It is worth recalling that SV40-contaminated live polio vaccines, as candidate vaccines, were tested during large field trials in some Latin American Countries, due to their potential for being naïve vaccines.

Overall, immunological data indicate that SV40 is circulating in humans inducing IgM, IgG including neutralizing antibodies, which can be detected in sera with a mean prevalence of ~20% in healthy individuals. Recent immunological data, despite being obtained with specific SV40 mimotopes, do not rule out the hypothesis that another polyomavirus, still unknown, closely related to SV40 is circulating in humans.

## Association of SV40 With Human Tumors

The hypothesis that SV40 might be associated to human malignancies has been investigated with a large number of molecular, immunological, and epidemiological studies (Table 2). This oncogenic PyV was previously associated with a broad range of tumor types including, malignant pleural mesothelioma (MPM) (80, 81, 227–233, 235, 238, 240–243, 256–258), bone (98, 215, 224), brain (212–214, 217, 219–221, 259, 260), lung (227, 234), thyroid (82, 244), pituitary (179), and urothelial (245) tumors, pleomorphic adenomas of parotid glands (83), choroid plexus tumors, and ependymomas in children (160). In addition, footprints from SV40 DNA have been detected in breast (84) and colon cancer specimens (222). Interestingly, DNA sequences belonging to SV40 have also been found in an AIDS patient with a cerebral lesion (216). More recently, a study conducted with an innovative analysis, known as RNA sequencing (RNA-seq), identified SV40 mRNAs, in tumor samples from low-grade glioma affected patients (219). Different lympho-proliferative disorders (85, 239, 254, 255), including non-Hodgkin's lymphoma (79, 247–251, 260) have also been associated with SV40 infection. It has been reported that the homologous and autologous implantation of SV40-transformed cells in humans caused the growth of nodules, thus suggesting that this PyV presents oncogenic capacity in humans (261). These studies support an association of human cancers with SV40. These results, obtained mainly by PCR, Southern blot hybridization, DNA sequencing, and immunohistochemistry (IHC) were confirmed by a multi-institutional study (80), but confuted by another group of investigators (262). Among SV40-positive tumor types, MPM has been detected as SV40-positive in many investigations (80, 81, 227–233, 235, 238, 240–243, 256–258, 263), while the mechanisms of SV40 oncogenesis have also been studied (9, 44, 258, 264, 265). Interestingly, when normal HMC were exposed to both SV40 and asbestos fibers, the transformation rate increased significantly compared to the controls (9). High exposition to asbestos fibers alone can cause progressive fibrosis (i.e., asbestosis) and in the worse cases, lung cancer (266) and MPM (9). Many studies reported the synergistic activities of SV40 and asbestos on MPM development in geographical areas with high levels of asbestos exposure and SV40-contaminated polio vaccines (9, 81, 187, 232, 236, 239). High prevalence of SV40 DNA sequences in MPM tissues reflects that relation to SV40-contaminated polio vaccines (9, 81, 187, 232, 236, 239). The majority of human cancers mentioned above correspond to tumors, which develop in rodents inoculated with SV40 and in SV40-transgenic mice.

**Table 2:** SV40-positive human tumors.

**Tumor type**	**No. of positive samples/total samples analyzed (%)**				**Technique(s)**	**References**
	**SV40 DNA**	**SV40 RNA**	**SV40 protein**	**Human serum Anti-SV40 Abs**		
**Solid tumors**						
Breast cancer	24/109 (22)	–	24/109 (22)	–	PCR, IHC	(84)
Breast cancer	–	–	–	12/78 (15)	ELISA	(211)
**Brain tumors**						
Astrocytoma	8/35 (23)	–	–	–	RA	(212)
Astrocytoma	8/17 (47)	–	–	–	PCR	(161)
Astrocytoma	–	–	11/15 (73)	–	IP, SS, WB	(213)
Astrocytoma	9/28 (32)	–	–	–	PCR, SB	(214)
Choroid plexus	10/20 (50)	–	4/5 (80)	–	PCR, SB, RA	(160)
Choroid plexus	10/20 (50)	–	–	–	PCR	(215)
Choroid plexus	5/6 (83)	–	–	–	PCR	(161)
Choroid plexus	6/16 (38)	–	–	–	PCR, SB	(214)
Cerebral lesion	1^*^	–	–	–	PCR, filter	(216)
Ependymoma	10/11 (91)	–	–	–	PCR	(215)
Ependymoma	4/13 (31)	–	–		PCR, SB, sequencing	(217)
Ependymoma	8/11 (73)	–	–	–	PCR	(161)
Ependymoma	–	–	8/8 (100)	–	IP, SS, WB	(213)
Ependymoma	10/11 (91)		3/6 (50)	–	PCR, SB, RA	(160)
Ependymoma	9/16 (56)	–	–	–	PCR, SB	(214)
Glioma	3/20 (15)	–	–		PCR, SB, sequencing	(217)
Glioblastoma	10/30 (33)	–	–	–	PCR	(161)
Glioblastoma	–	–	4/8 (50)	–	IP, SS, WB	(213)
Glioblastoma	9/46 (20)	–	–	–	PCR, SB	(214)
Glioblastoma	–	–	–	15/44 (34)	ELISA	(218)
Gliosarcoma	4/20 (20)	–	–	–	PCR, SB	(214)
Low grade Astrocytoma	13/22 (58)	–	–	–	PCR, SB	(214)
Low grade Glioma	–	40/172 (23)	–	–	RNA-seq	(219)
Meningioma	1/7 (14)	–	–	–	PCR	(161)
Meningioma	1^*^	–	–	–	PCR, sequencing	(220)
Various brain tumors^Δ^	–	11/32 (34)	–	–	*In situ* hybridization	(221)
Colon cancer	6/94 (6)	–	–	–	PCR, qPCR	(222)
Nasopharyngeal carcinoma	–	–	–	16/64 (25)	ELISA	(223)
Osteosarcoma	5/10 (50)	–	–	–	PCR, sequencing	(224)
Osteosarcoma	9/35 (26)	–	–	–	PCR, SB	(98)
Osteosarcoma	54/160 (34)	–	–	–	PCR	(215)
Osteosarcoma	–	–	–	24/55 (44)	ELISA	(225)
Osteosarcoma	–	–	–	87/249 (35)	ELISA	(190, 280)
Osteosarcoma	143/277 (52%)	–	–	–	PCR	(226)
**Thoracic tumors**						
Adenocarcinoma	7/15 (47)	–	–	–	PCR, SB	(227)
Bronchopulmonary carcinoma	18/63 (29)	–	0/16 (0)	–	PCR, SB, IHC	(227)
Malignant mesothelioma	10/21 (48)	–	0/15 (0)	–	PCR, SB, IHC	(227)
Malignant mesothelioma	3/30 (10)	–	–	–	PCR, sequencing	(228)
Malignant mesothelioma	5/5 (100)	5/6 (83)	–	–	PCR, qPCR	(229)
Malignant mesothelioma	29/48 (60)	–	13/16 (81)	–	PCR, IHC	(230)
Malignant mesothelioma	12/10 (83)	–	12/10 (83)	–	PCR, sequencing, IHC	(80)
Malignant mesothelioma	4–26/26 (15–100)^#^	–	–	–	PCR	(231)
Malignant mesothelioma	3/19 (16)	–	–	–	QPCR	(232)
Malignant mesothelioma	15/25 (60)	–	15/25 (60)	–	PRINS, IHC	(233, 234)
Malignant mesothelioma	4/11–0/9 ^γ^	–	–	–	PCR, SB	(235)
Malignant mesothelioma	8/19 (42.1)	–	–	–	PCR	(236)
Malignant pleural mesothelioma	–	–	–	25/97 (26)	ELISA	(237)
Malignant pleural mesothelioma	20/40 (50)	–	–	–	PCR, sequencing	(238, 239)
Malignant pleural mesothelioma	2/35 (6)	–	–	–	QPCR	(240)
Malignant pleural mesothelioma	8/18 (45)	–	0/18 (0)	–	PCR, sequencing, IHC	(241)
Malignant pleural mesothelioma	4/9 (45)	–	–	–	PCR	(242)
Malignant pleural mesothelioma	–	–	9/45 (20)	–	IHC	(81)
Pleural/peritoneal mesotheliomas	67/118 (57)	–	–	–	PCR, sequencing	(243)
Various lung cancers^Σ^	12/35 (34)	–	–	–	PCR, SB	(227)
Thyroid tumor	3/69 (4)	–	–	–	PCR, SB, sequencing	(244)
Thyroid tumor	12–19/19–29 (66–100)^#^	17/24 (71)	11/17	–	PCR, SB, IHC, Sequencing, qPCR	(82)
Pleomorphic adenoma	28/45 (62)	–	26/28 (93%)	–	PCR, IHC	(83)
Pituitary tumor	26/30 (87)^§^	–	0/18 (0)	–	PCR, SB, IHC	(179)
Urothelial tumor	–	–	1^*^		IHC	(245)
Urothelial tumor	6/18 (42.1)	–	–	–	PCR	(236)
Uveal Melanoma	–	–	–	16/48 (33)	ELISA	(246)
**Liquid tumors**						
Hodgkin's lymphoma	7/43 (16)	–	2/7 (28)	–	PCR, sequencing, IHC	(79)
Hodgkin's lymphoma	16/54 (30)	–	–	–	Multiplex Nested PCR	(238, 247)
Hodgkin's lymphoma	2/19 (10)	–	2/2 (100)	–	PCR, SB, IHC, sequencing	(85)
Non-Hodgkin's lymphoma	8/58 (14)	–	–	–	PCR, SB	(182)
Non-Hodgkin's lymphoma	3/29 (10)	–	–	–	PCR	(172, 248)
Non-Hodgkin's lymphoma	17/42 (40)	–	–	–	PCR	(249)
Non-Hodgkin's lymphoma	6/36 (17)	–	–	–	PCR, sequencing	(247, 250)
Non-Hodgkin's lymphoma	12/55 (22)	–	12/12 (100)	–	PCR, IHC	(251)
Non-Hodgkin's lymphoma	11/79 (14)	–	3/11 (27)	–	PCR, sequencing, IHC	(79)
Non-Hodgkin's lymphoma	–	–	–	36/89–26/61 (40,41,42,43)^γ^	ELISA	(252)
Non-Hodgkin's lymphoma	–	–	–	55/150–37/104 (37–36)^γ^	ELISA	(18, 195, 197, 253)
Non-Hodgkin's lymphoma	85/158 (54)	–	–	–	Multiplex Nested PCR	(238, 239)
Non-Hodgkin's lymphoma	28/106 (26)	–	28/28 (100)	–	PCR, SB, IHC, sequencing	(85)
Non-Hodgkin's lymphoma	63/108 (56)	–	–	–	PCR	(254)
Non-Hodgkin's lymphoma	38/60 (63)	–	–	–	PCR, qPCR	(255)
Leukemia	22/54 (41)	–	–	–	Multiplex Nested PCR	(238, 239)
Leukemia	16/48 (30)	–	–	–	PCR	(249)
Various Leukemias^¥^	16/19 (84)	–	–	–	PCR, qPCR	(255)

* Case reports;

Δ Angiofibroma, astrocytoma, metastatic brain tumors, meningiomas, neurinomas, oligodendrogliomas;

# Different primer set;

γ Two different cohorts;

Σ Pleomorphic carcinoma, Neuroendocrine carcinoma, Squamous cell carcinoma, others not specified

§ Polyomaviral primers that hybridized to SV40 and BKPyV internal probes;

¥ *Bcell acute lymphoblastic leukemia, B-cell precursor acute lymphoblastic leukemia, T-cell acute lymphoblastic leukemia. IP, Immunoprecipitation; SS, Silver staining; WB, Western blot; PCR, Polymerase chain reaction; PRINS, Primed in situ assay (DNA detection); qPCR, real time quantitative PCR; IHC, Immunohistochemistry; RA, restriction analysis*.

It appears that in the same kind of tumor, prevalence of SV40 sequences differs in distinct geographical areas. For example, it has been reported that in the U.S. and Europe 20–83% of MPM tested SV40-positive (80, 228, 230, 235, 236, 249), while sequences of this PyV in MPM from Turkey and Austria have never been detected (235, 267), or detected with low frequency, as in Sweden (228). Similarly, the prevalence SV40 DNA detected in bone tumors is different for example in Hungary (74%) and Germany (24%) (226). In addition, SV40-positive MPMs were found in two different studies in Japan with a prevalence ranging between 6 and 44% (241, 268), whereas another investigation conducted in a cohort of Vietnamese MPM patients detected 20% SV40-positive tumors (81).

The association between SV40 and human tumors is based on results obtained by many investigators (80, 98, 213, 218, 220, 225, 227, 230, 234, 255, 256). Most of these studies detected SV40 DNA sequences, using qualitative PCR techniques, in tumor specimens. However, these assays do not provide the quantification of SV40 DNA as a copy number, nor investigated the physical state of the viral DNA, i.e., integrated and/or episomal (96, 98). In other studies SV40 mRNA and/or Tag/tag oncoproteins were analyzed in tumors (9, 68, 81, 83, 160, 219), or quantified the amount of SV40 DNA sequences via qPCR methods (240, 268, 269). The majority of these qualitative/quantitative assays (Table 2) were carried out targeting different SV40 Tag sequences. Together with these sequences, other SV40 regions were PCR analyzed, including the control region and late gene sequences (181, 220). These data indicate that SV40 DNA regions detected in human tumors were not due to PCR contaminations with recombinant plasmids carrying SV40 Tag sequences. In addition, in studies based on IHC staining, the localization of the SV40 Tag in the SV40-infected cell is shown (79–85). Studies carried out on SV40 Tag-positive cells demonstrated that the cell transformation is related to the activation of specific autocrine/paracrine loops. In this context, different growth factors and their receptors were analyzed, such as HGF and its receptor (HGFR or Met) (44), the vascular endothelial growth factor (VEGF) and its receptor (VEGFR) (270, 271) as well as (IGF-1) and its receptor, in T-antigen-mediated growth (272, 273). Concordant data were reported on the ability of the SV40 Tag to induce growth factor receptor/growth factor loops, which in turn stimulates cell-cycle progression into the S phase. These results suggest that a few cells, found to be SV40-positive, induce SV40-negative cells on the microenvironment toward the transformation. The SV40-infected cells have also been determined by several *in vitro* studies (56, 58–65). Different hypotheses have been formulated on the mechanisms of SV40 carcinogenesis in human, including the “hit and run mechanism” which has been investigated in transgenic mice (274). However, this hypothesis cannot be proven in humans. Most of the reports mentioned above are related to association studies in which the causative role of SV40 in inducing human tumors cannot be proven. It is plausible that this PyV may act as a co-factor during the tumorigenic process, either during the early phase of oncogenesis or in the late phase of tumor progression. Indeed, as for other viruses (17), in normal physiological conditions, the immune system counteracts the oncogenic potential of SV40 (194) whose infection could be acquired early in life (190, 193). However, in certain host conditions, such as in immunocompromised individuals, SV40 may exert its oncogenic activities. It is also possible that SV40 acts in a late phase when the cell is already transformed. In these pre-neoplastic cells SV40 Tag/tag expression could favor full transformation. Further studies may clarify this important aspect.

Some studies have reported negative data on an association between human tumors and SV40 (235, 263, 274–277). These investigations have shown neither viral DNA (or SV40 DNA at low prevalence) nor viral oncoproteins and miRNAs in the same tumor types found to be SV40-positive in other studies (240, 248, 275–278). This is an important issue that has fueled the controversy about SV40 in humans and in human tumors (262). It is important to point out, however, that a SV40 prevalence of about 5% was shown in those studies, even if it was considered statistically no-significant in considering SV40 a risk factor for human cancers (9, 240, 262). Another relevant issue was highlighted by Lopez-Rios et al. who evidenced the risk of false-positive PCR results accountable by plasmids carrying SV40 sequences circulating in common laboratories, thereby creating overly-high positive rates (262). Negative results have also been published in another independent report from Aaronson and colleagues, reaching similar conclusions (279). Indeed, this study shows no evidence for Tag antigen expression in a series of MPM tumors and derived cell lines (279). For this reason, over time, molecular techniques/protocols have been used ever-more carefully in order to prevent false-positive results, while still revealing any case evidence of SV40 infection in humans and human cancers (270, 276). The different assays employed to isolate DNA from tumor tissues affecting the results is also considered as an explanation for this discrepancy (79). Indeed, several commercial kits prevent the isolation of SV40 DNA that is lower than 5.2 kb in length, i.e., too small. Furthermore, SV40 DNA can be amplified with certain sets of primers but not with others (160, 230). In summary, further studies with new approaches are needed to clarify these conflicting results and to address the role of SV40 in human cancers.

To better elucidate these controversies, novel indirect ELISA tests employing synthetic peptides as mimotopes/specific SV40 antigens were set up (191, 192). These immunological and specific assays established that anti-SV40 antibodies can be revealed in human sera from patients affected by different tumors (211, 218, 223, 225, 237, 246, 252, 253, 280, 281), of the same kind found to be SV40-positive by molecular biology techniques. A significantly higher SV40 antibody prevalence was detected in sera from MPM, glioblastoma multiforme, osteosarcoma, and non-Hodgkin's lymphoma patients compared to age-/gender-matched healthy subjects (218, 225, 237, 252, 253, 280).

## Association of SV40 With Human Non-Malignant Diseases

Many reports have suggested that the kidney could be the main site for SV40 latency in humans as it occurs in monkeys, that are the natural animal host (1, 189). For this reason, an association between SV40 infection and kidney-related diseases was investigated. DNA sequences from this PyV were found in renal tubular epithelial cells nuclei, PBMCs and renal biopsies derived from patients affected by focal segmental glomerulosclerosis, thus suggesting the possible involvement of SV40 in kidney diseases (184). This assumption has been further strengthened through the isolation of SV40 virions in co-cultured urine sediment cells from a nephropathy-affected patient with CV-1 cells, which are SV40 permissive cells derived from monkeys (183). Furthermore, molecular evidence that SV40/BKPyV co-infection occurs in patients with post-transplantation interstitial nephritis has also been reported, suggesting that SV40 may contribute to this disease after the renal transplant in cooperation with BKPyV (183). Other studies indicate that SV40 seems to be associated with neurological diseases, including multiple sclerosis (281–283). DNA sequences from SV40 have also been detected in allografts from immunocompromised pediatric renal transplant recipients and in the kidneys of young adult lung-transplant patients (162, 284). Therefore, it is reasonable to propose that a weak immune system, typical of transplant recipients subjected to iatrogenic immunosuppression, could be a risk factor for SV40 infection, as for other PyVs (17).

## Conclusions

SV40 infection in different human populations worldwide has been reported by many groups (160–164). Indeed, SV40 DNA sequences were detected in normal tissues, such as PBMCs (161, 182, 187), leukocytes from organ and blood donors (180, 181), and pituitary tissues (179). Specific immunological assays identified IgG and IgM antibodies against SV40 VPs and Tag in sera from normal children, adults and elderly subjects (190, 191, 193, 194). In addition, SV40 neutralizing antibodies were detected, in different investigations, using the plaque reduction assay, which is a high specific test demonstrating that the SV40 infection occurred in subjects/patients (203, 210, 285). These data cumulatively indicate that SV40, or a closely related polyomavirus, is circulating in humans. Conflicting results have been published on the association between different human tumors and SV40. Although this oncogenic PyV was previously associated with a broad range of tumor types including brain (213, 220, 234) and bone tumors (98), MPMs (80, 81, 227, 230), and different lymphoproliferative disorders (85, 239, 249, 254), other investigators reported negative results when analyzing the same tumor types (238, 275–279). In addition, these studies are mainly based on the detection of viral DNA. Other works carried out in tumor specimens investigated, to a lesser extent, the viral DNA status (integrated or/and episomal), mRNA, and the expression of viral oncoproteins (84, 98, 213, 219). Immunological studies detected specific antibodies against SV40 in sera from tumor affected patients (218, 225, 237, 252, 280).

The existence of an SV40-like human polyomavirus, which is still unknown, cannot be ruled out. Recently, a new lymphotropic polyomavirus (HPyV9) was identified in humans (286). It turned out that HPyV9 has great homology with the monkey LPyV, which has been known since the 1960s (287).

The role of SV40 in human tumors, if any, remains to be proven. This is an important issue and certainly deserves further attention with detailed and innovative investigations.

## Author Contributions

JR: data collection from the literature, writing, and figure preparation. EM and IB: data collection from the literature and figure preparation. MT and FM: manuscript revision.

### Conflict of Interest Statement

The authors declare that the research was conducted in the absence of any commercial or financial relationships that could be construed as a potential conflict of interest.
